# Impaired hemodynamic forces assessed by routine CMR and its determinants in different duration T2DM patients with normal LV function and myocardial strain

**DOI:** 10.3389/fcvm.2025.1460094

**Published:** 2025-01-31

**Authors:** Guozhu Shao, Yukun Cao, Yue Cui, Hongyan Li, Heshui Shi

**Affiliations:** ^1^ Department of Radiology, Union Hospital, Tongji Medical College, Huazhong University of Science and Technology, Wuhan, China; ^2^ Hubei Provincial Clinical Research Center for Precision Radiology & Interventional Medicine, Wuhan, China; ^3^ Hubei Province Key Laboratory of Molecular Imaging, Wuhan, China; ^4^ Department of Nuclear Medicine, Zhongnan Hospital of Wuhan University, Wuhan University, Wuhan, Hubei, China

**Keywords:** type 2 diabetes mellitus, hemodynamic forces, myocardial strain, late gadolinium enhancement, cardiac magnetic resonance imaging

## Abstract

**Background:**

Early detection of subclinical myocardial dysfunction in asymptomatic patients with type 2 diabetes mellitus (T2DM) is essential before overt changes in left ventricular ejection fraction (LVEF) and myocardial strain occur. The objective of this study is to quantitatively assess hemodynamic forces (HDFs) using a rigorous mathematical model based on conventional cine cardiac magnetic resonance (CMR) images in patients with T2DM, and investigate their correlation with late gadolinium enhancement (LGE) and duration of diabetes.

**Methods:**

We recruited 63 T2DM patients and 50 healthy volunteers to undergo contrast-enhanced CMR examinations. T2DM patients were divided into three groups according to the course of disease: early, middle and later stage (time <5 years, 5 ≤ time <10 years, time ≥10 years, respectively). LV deformation parameters, global circumferential strain (LVGCS), radial strain (LVGRS), longitudinal strain (LVGLS) and HDFs parameters such as longitudinal (apical-basal/A-B), transversal (lateral-septal/L-S) HDF strength (RMS) were measured and compared among the three groups.

**Results:**

Compared with healthy volunteers, no significant differences in LV function and strains were observed (*P* > 0.05), while HDF Strength (RMS) L-S (%) were significantly higher in T2DM patients (*p* < 0.001). LVGLS was significantly decreased in late T2DM patients (*p* < 0.05), but HDF Strength (RMS) L-S (%) was significantly increased compared with early T2DM patients. Both HDF Strength (RMS) L-S (%) and HDF Strength (RMS) A-B (%) value were independently related to the extent of LGE (*β* = 0.435, *p* = 0.001; *β* = *−0.329*, *p* = 0.006, respectively). In addition, HDF Strength (RMS) L-S (%) was also independently correlated with insulin treatment(*β* = 0.291, *p* = 0.013).

**Conclusions:**

HDF analysis can provide valuable insights into subclinical myocardial dysfunction prior to changes in ejection fraction and myocardial strain, suggesting that HDF analysis may be a potential early marker of subclinical myocardial dysfunction. LVGLS damage is gradually obvious with the prolongation of diabetes duration in T2DM patients. HDFs parameters are associated with the extent of LGE, and the transversal component of HDF increased with the duration of diabetes.

## Background

Diabetes mellitus (DM) is a group of metabolic diseases and is a major epidemic disease in this century (1). Diabetic cardiomyopathy (DCM) refers to structural and functional abnormalities of the heart that are independent of coronary artery disease and hypertension and can lead to heart failure, which is an important risk factor for cardiovascular disease morbidity and mortality in diabetic patients (2). DCM is different from other cardiomyopathies, because its pathogenesis is complex, involving a variety of factors and pathogenic mechanisms, and the onset is more insidious, which brings great difficulty to clinicians in early detection and diagnosis. Thus, early detection of subclinical myocardial dysfunction and timely intervention are crucial to the management of asymptomatic patients with type 2 diabetes mellitus (T2DM).

At present, echocardiographic particle image velocimetry can be used to assess hemodynamic forces (HDFs) evaluation (3), but the demand for contrast agent infusion and high quality images recorded at high frame rates resulted in poor applicability. Four-dimensional flow cardiac magnetic resonance imaging (CMR) is a highly reproducible method and is considered to be the reference standard for HDF measurement, however, it is limited by the long scanning time, complex operation and high cost. Nowadays, a simplified model mathematical model was based on the first principle of fluid dynamics (4) that allows estimating HDF by routine conventional cine CMR images shown to possibly be capable of overcoming the above weakness through the knowledge of left ventricular (LV) geometry, endocardial tissue movement, and areas of the aortic and mitral orifices, without knowing the velocity field inside the LV, making it more readily accessible for routine. The interaction of the heart valves, great vessels, and myocardium creates a ventricular pressure gradient (IVPG) that drives intraventricular blood flow. HDFs analysis is the global value of IVPG integrated over the ventricular volume (5), it represents a novel approach to quantify IVPG. Many studies have shown that there are subtle changes in diastolic function, myocardial strain, and myocardial perfusion in patients with T2DM (6, 7). However, HDFs parameters derived from conventional CMR is very important for early detection of subclinical myocardial dysfunction when LV volume and myocardial deformation index are still intact in asymptomatic T2DM patients, and such studies have not been reported.

In our previous study, we found that there were no significant changes in LV function and myocardial strain parameters in T2DM patients compared healthy persons (8). Given that HDF analysis can earlier detect subclinical myocardial dysfunction in T2DM patients before overt changes in LVEF and myocardial strain occur (9), therefore, in this study, the objective of this study is to quantitatively assess hemodynamic forces (HDFs) using a rigorous mathematical model based on CMR images in T2DM patients, and investigate their correlation with the extent of late gadolinium enhancement (LGE) and duration of diabetes.

## Methods and materials

### Study population

**Figure 1:**
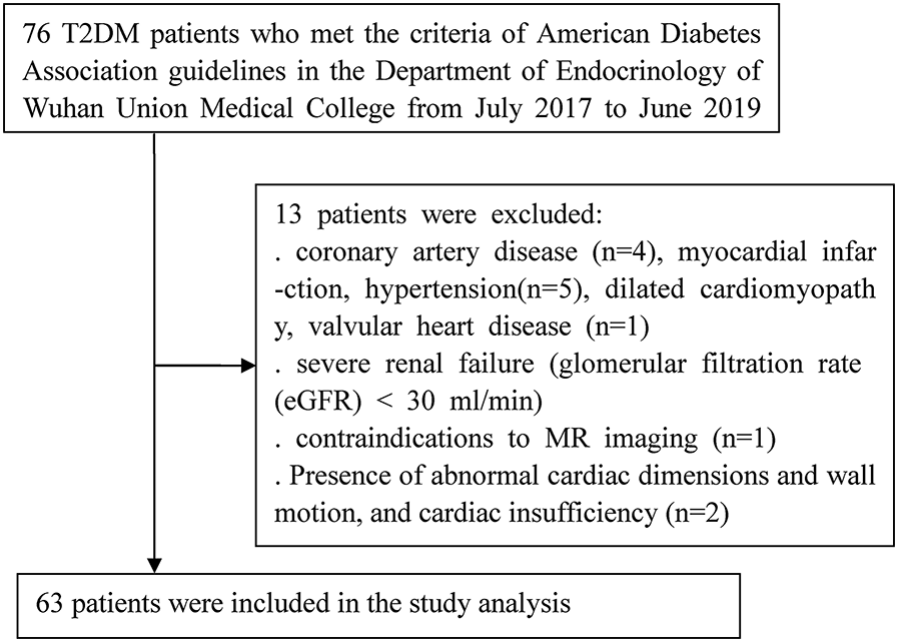
Flow chart of subjects included in the study.

From July 2017 to June 2019, 76 consecutive T2DM patients required to meet the current American Diabetes Association guidelines (10) were prospectively recruited from the department of endocrinology at Wuhan Union Hospital. T2DM patients. The exclusion criteria encompassed clinical evidence of coronary artery disease, myocardial infarction, hypertension, dilated cardiomyopathy, valvular heart disease, severe renal failure [glomerular filtration rate (eGFR) < 30 ml/min], contraindications to MR imaging, the presence of abnormal cardiac dimensions and wall motion, and cardiac insufficiency (LVEF < 50%). Finally, 63 T2DM patients and 50 healthy controls were enrolled in this study (Figure 1). For comparison, 50 healthy volunteers matched by age, sex and BMI from the community with no history of cardiac disease or diabetes mellitus, hypertension, hyperlipidemia. All recruited normal volunteers underwent physical examination with normal results. The exclusion criteria includedrenal dysfunction (glomerular fltration rate < 30 ml/min), active pregnancy, contraindications for MRI, left ventricular hypertrophy, presence of abnormal cardiac dimensions, global or regional LV wall motion abnormalities, valvular stenosis or regurgitation or myocardiall LGE during the MRI examination. This study was registered at the Chinese Clinical Trials Registry Center registration number (ChiCTR2000039816), and was approved by the Ethics Committee of Tongji Medical College, Huazhong University of Science and Technology [No. (2019)S878]. Each participant provided written informed consent.

### Anthropometric and biochemistry

We collected the sex, age, height, body weight, and blood pressure of all subjects. The duration of diabetes was reported by the patients. Blood samples were collected after overnight fasting before the MRI examination. Laboratory tests, including glycosylated hemoglobin (HbA1c), microalbuminuria (MA), serum glucose, creatinine, triglycerides (TG), total cholesterol (TC), blood urea nitrogen (BUN), high-density lipoprotein cholesterol (HDL-C), and low-density lipoprotein cholesterol (LDL-C), as well as medication history, were performed for all patients using electronic medical records.

### CMR scanning protocol

All subjects underwent a standard CMR examination with a 1.5 T MR (MAGNETOM Aera, Siemens Healthcare, Erlangen, Germany) with a dedicated two-element cardiac-phased array coil. A balanced steady-state free procession (b-SSFP) sequence was used to obtain cine images, including the acquisition of three long-axis (two-, three-, and four-chamber) and short-axis (coverage from the the mitral valve to the apex.) slices. The cine image parameters were as follows: repetition time (TR)/echo time (TE), 2.9/1.2 ms; slice thickness, 6 mm; flip angle, 80°; FOV, 360 × 270 mm^2^; matrix, 144 × 256 pixels; voxel size, 1.3 × 1.3 × 8.0 mm^3^; and scanning time, the duration of 11 heartbeats. LGE imaging of the long- and short-axes for the LV was performed 10–15 min after a bolus injection of intravenous gadolinium-diethylenetriamine pentaacetic acid (DTPA) (0.2 mmol/kg, Magnevist; Bayer Healthcare; Germany) with a phase-sensitive inversion recovery (PSIR) sequence. LGE was defined as the area of signal intensity five standard deviations above the mean intensity of the normal myocardium on the LGE short axis images (11). The LGE imaging parameters were as follows: repetition time, 12.44 ms; echo time, 1.19 ms; inversion recovery time, 300 ms; flip angle, 40°; slice thickness, 8 mm; field of view, 360 × 270 mm^2^; and matrix, 256 × 192.

### CMR imaging analysis

All CMR images were transferred to an off-line workstation and processed with a commercial cardiovascular postprocessing software(Medis Medical Imaging Systems, Leiden, the Netherlands). LV function parameters, including LV end-diastolic volume (EDV), end-systolic volume (ESV), stroke volume (SV), and ejection fraction (EF) were calculated by manually tracing LV endocardial and epicardial contours in serial short-axis slices at the end-diastolic and end-systolic phases.

LV myocardial strain analysis and HDF measurements were performed using the Medis QStrain package. Endocardial and epicardial contours with the exclusion of the papillary muscles were delineated in the end-systole and end-diastolic phase of 2-, 3-, 4-chamber, and short-axis cine images. The left ventricular global longitudinal (GLS), circumferential (GCS) and radial strain (GRS) were calculated by automatically tracking the contours in each cardiac cycle, as we used in our previous study (12).

**Figure 2:**
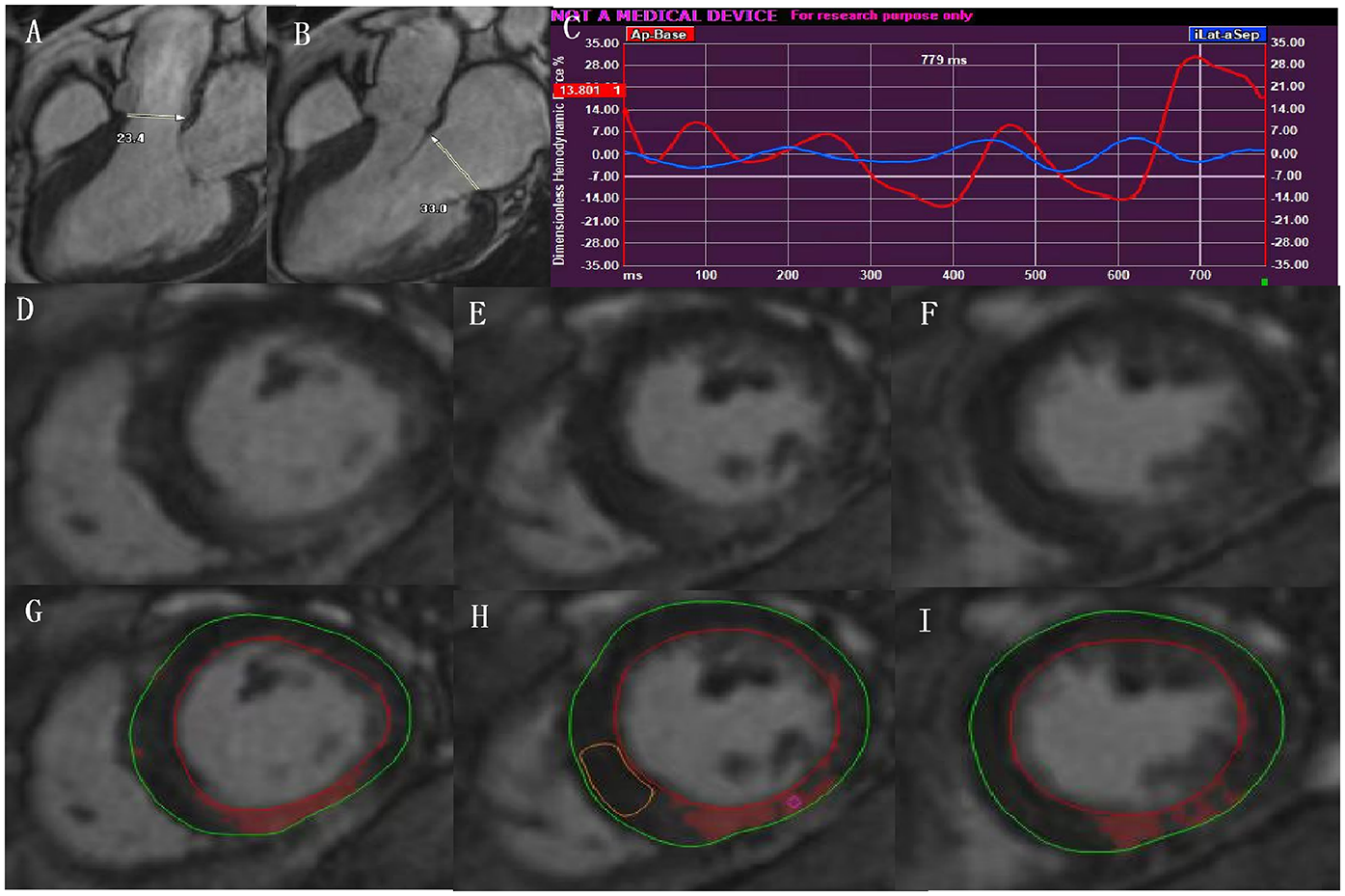
In the context of the endocardial border tracking used for LVGLS calculation, HDF can be acquired by measurements of the aortic and mitral diameter in the LV 3-chamber view **(A,B)**, and with corresponding HDF curves **(C)**; representative contours of LV LGE images from basal-apical segments in a patient with type 2 diabetes **(D–F)**. The same images showing quantification of LGE using the 5SD thresholding method **(G–I)**. LVGLS, left ventricular global longitudinal strain; HDF, hemodynamic forces; LGE, late gadolinium enhancement.

In the context of the endocardial border tracking used for LVGLS strain calculation, the maximum diameters of the aortic valve in the end-systole phase and the maximum diameters of the mitral valve in the end-diastolic phase were measured in the 3-chamber view. Then, the values of HDFs can be derived by the measured maximum diameter of aortic valve and mitral valve using the mathe-matical model integrated into the dedicated Medis software (13) (Figures 2A,B), with corresponding HDF curves (Figure 2C).

The diameters of the aortic and mitral valves were assessed in the 3-chamber view.

The HDFs were then quantified in the longitudinal (apical-basal/A-B) and main transversal (lateral-septal/L-S) directions. To enhance comparability among patients with varying LV sizes, the HDFs were normalized with the LV volume and expressed as a percentage of gravity acceleration. These normalized forces were expressed in dimensionless form and represented the integral of pressure gradient in the LV cavity.

The HDFs in the longitudinal and main transversal directions were described as root mean square (RMS), and represent the mean amplitude of longitudinal and transverse force during entire heartbeat respectively.

For the quantification of the extent of LGE, we imported the whole LV short-axis slices of the LGE images into the software (Figures 2D-F), and manually delineated the LV endocardial and epicardial contours. The areas of delayed myocardial enhancement was defined as a signal intensity threshold of >5 standard deviation (SD) above the mean signal intensity of the normal myocardium (14) (Figures 2G–I).

### Reproducibility analysis of LV strain and HDF index

The reproducibility of LV myocardial strain and HDF index measurements were analyzed by two investigator who was blinded to patient/control status. One observer (GZ. S) measured LV global myocardial strain in 40 random subjects (including 20 T2DM patients and 20 controls) twice within one month. A second observer (HY. L), who was blinded to the results of the first observer and clinical data, reperformed the measurements to assess the interobserver variability.

### Statistical analysis

All data were statistically analyzed using standard statistical software (SPSS 21.0 for Windows, IBM, Chicago, IL, USA). The Shapiro–Wilk test was performed to evaluate data for normality and Levene's test for homogeneity of continuous variables. The continuous normally distributed data were expressed as means ± standard and non-normally distributed variables were expressed as medians with interquartile ranges. Categorical variables were presented as frequencies (percentages). Differences in continuous variables between T2DM and Control were compared using an independent sample Student's t test. The myocardial strain and HDF index among T2DM patients (early, middle and later stage) were compared by Analysis of variance (ANOVA) followed by Bonferroni's *post hoc*-test (normally distributed variables) or the Kruskal–Wallis rank test (nonparametric variables) where appropriate. Pearson's or Spearman's correlation test was applied for the assessment of the HDF index and all candidate variables. All candidate variables (*p* < 0.1 in the univariate linear regression analysis and without collinearity) were selected for entry into the multiple stepwise regression model. The intraclass correlation coefficient (ICC) was used to evaluate both inter- and intraobserver variability. A *p* value < 0.05 (two-tailed) was considered significant.

## Results

### Clinical characteristics of the study population

The general characteristics of the study subjects are summarized in Table 1. No differences were observed with respect to sex, age, BMI, and blood pressure between the patients and the controls.

**Table 1:** Clinical characteristics of study subjects.

	T2DM (*n* = 63)	Control (*n* = 50)	*P* value
Age (years)	52.5 ± 8.3	53.1 ± 5.8	0.352
Male, *n* (%)	34 (54.0)	26 (52)	0.763
BMI (kg/m^2^)	24.1 ± 3.7	24.2 ± 3.0	0.856
Diabetes duration (y)	9 (5–12)	–	–
SBP (mmHg)	127 ± 12	123 ± 10	0.321
DBP (mmHg)	77 ± 9	78 ± 8	0.278
BUN (mmol/L)	5.4 ± 1.5	–	–
Creatinine (μmol/L)	68.0 ± 14.0	–	–
Total cholesterol (mmol/L)	4.5 ± 0.9	–	–
Triglycerides (mmol/L)	1.4 ± 1.1	–	–
HDL-C(mmol/L)	1.4 ± 0.4	–	–
LDL-C (mmol/L)	2.6 ± 0.7	–	–
FPG (mmol/L)	8.3 ± 3.1	–	–
Hemoglobin A1C (%)	8.9 ± 2.3	–	–
Microalbuminuria (MA)	12.6 ± 5.8	–	–
Diabetic complication, *n* (%)			
Retinopathy	22 (34.9)	–	–
Neuropathy	15 (44.1)	–	–
Peripheral vascular disease	12 (19.0)	–	–
Hypoglycemic medication, *n* (%)			
Insulin	33 (52.4)	–	–
Metformin	29 (46.0)	–	–
Sulphonylurea	13 (20.6)	–	–
Other medication, *n* (%)			
Statin	25 (39.7)	–	–
Aspirin	17 (27.0)	–	–
ACEI	21 (33.3)	–	–
Diuretics	5 (7.9)	–	–
Calcium channel blockers	5 (7.9)	–	–
β-blockers	14(22.2)	–	–

All data expressed as mean ± SD, percentage (number of participants), or median (interquartile range), as appropriate.

T2DM-HT, type 2 diabetes mellitus-hypertension; BMI, body mass index; HR, heart rate; SBP, systolic blood pressure; DBP, diastolic blood pressure; BUN, blood urea nitrogen; HDL-C, high-density lipoprotein cholesterol; LDL-C, low-density lipoprotein cholesterol; FPG, fasting plasma glucose; ACEI, angiotensin-converting enzyme inhibitor.

### MRI characteristics of the study population

**Figure 3:**
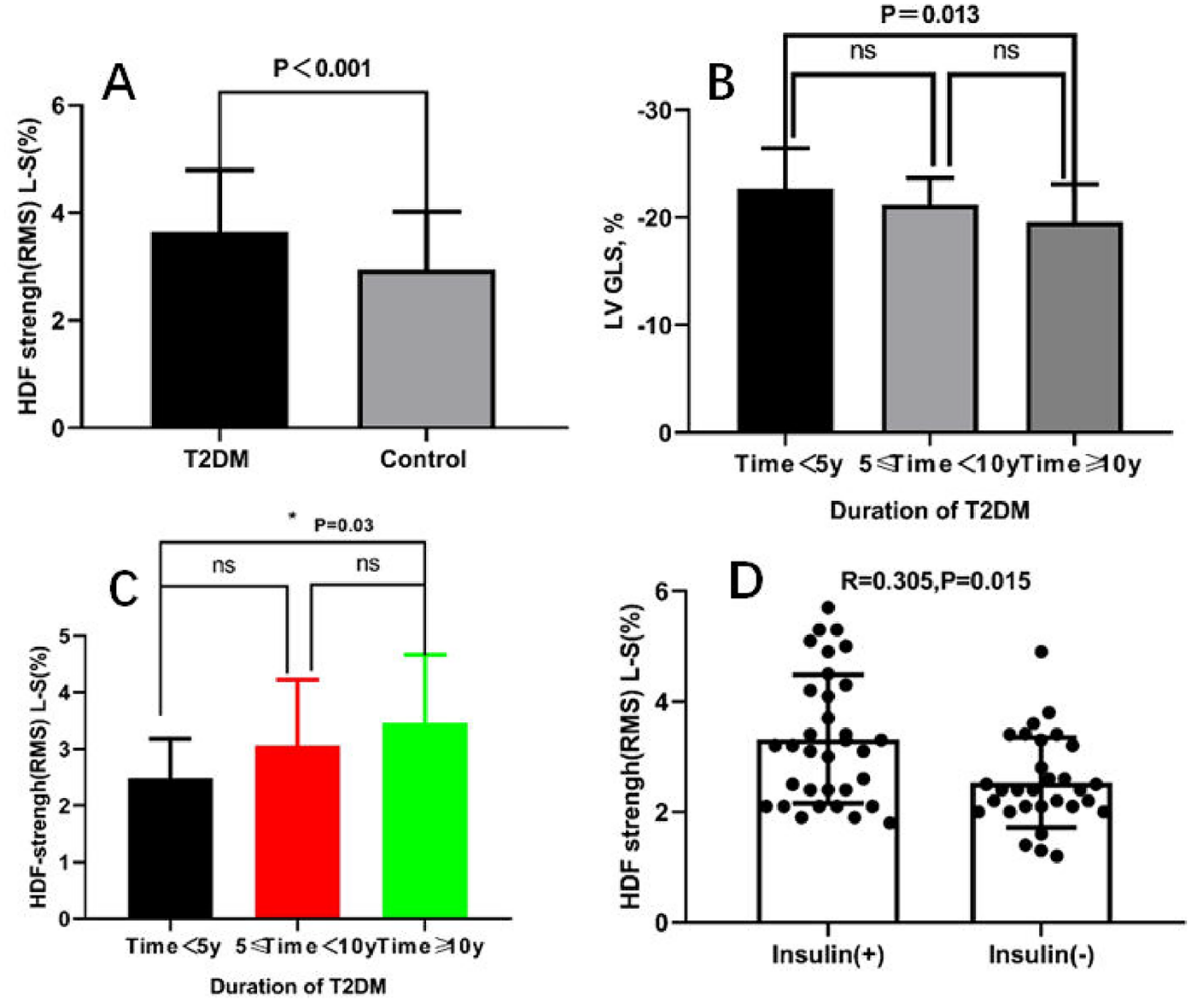
Comparison of t HDF strength (RMS) L-S vaule between T2DM and control **(A)** comparison of LVGLS and HDF strength (RMS) L-S vaule among different course of diabetic patients **(B,C)**; the relationship between HDF strength (RMS) L-S and insulin treatment in diabetic patients **(D)**. T2DM, type 2 diabetes mellitus; LVGLS, left ventricular global longitudinal strain; HDF, hemodynamic forces; RMS, root mean square.

Table 2 presents the baseline MRI characteristics of the patients with T2DM and the controls. No significant differences were observed in the LV function, myocardial strains between T2DM patients and the controls (*p* > 0.05), while HDF Strength (RMS) L-S (%) were significantly higher in T2DM patients (*p* < 0.001) (Figure 3A).

**Table 2:** MRI characteristics of study population.

	T2DM (*n* = 63)	Control (*n* = 50)	*P* value
LV function and strains			
LVEDV index (ml/m^2^)	58.5 ± 16.7	62.1 ± 12.4	0.532
LVESV index (ml/m^2^)	26.2 ± 6.4	27.1 ± 6.0	0.765
LVEF (%)	54.8 ± 5.1	57.3 ± 4.9	0.412
LVM index (g/m^2^)	53.6 ± 5.2	54.5 ± 5.9	0.545
LVGRS (%)	74.0 (62.1, 86.5)	73.3 (61.5, 82.4)	0.501
LVGCS (%)	−21.0 ± 4.3	−21.6 ± 2.8	0.367
LVGLS (%)	−21.4 ± 3.7	−22.0 ± 2.6	0.289
HDFStrength(RMS)-entire heartbeat			
HDF Strength(RMS)A-B(%)	18.6 (15.5, 22.3)	19.7 (17.7, 24.3)	0.593
HDFStrength(RMS) L-S (%)	3.6 (2.8, 4.4)	2.6 (2.1, 3.4)	<0.001
LV- myo-LGE(%)	4.7 (0, 8.7)	–	–

T2DM-HT, type 2 diabetes mellitus with hypertension; LVEDV, left ventricular end-diastolic volume; LVESV, left ventricular end-systolic volume; SV: stroke volume; LVEF, left ventricular ejection fraction; LVM, left ventricular mass; LVGRS, left ventricular global radial strain; LVGCS, left ventricular global circumferential strain; LVGLS, left ventricular longitudinal strain; A-B means in apical-basal direction; L-S means in apical-basal direction. HDF, hemodynamic force, RMS, root mean square; LV-myo-LGE, left ventricular myocardical late gadolinium enhancement.

Table 3 presents MRI characteristics of T2DM stratified according to duration of diabetes mellitus. The LVGLS value was significantly decreased in late T2DM patients (−22.7 ± 3.7 vs. −19.6 ± 3.5, *p* < 0.05) (Figure 3B), but HDF Strength (RMS) L-S (%) was significantly increased compared with early T2DM patients (3.5 ± 1.2 vs. 2.5 ± 0.7, *p* < 0.05) (Figure 3C). There was no significant difference in LV-myo-LGE (%) among the early, middle and later stages of T2DM.

**Table 3:** MRI characteristics of T2DM stratified according to duration of diabetes mellitus.

	Time < 5y (*n* = 14)	5 ≤ Time < 10y (*n* = 22)	Time ≥ 10y (*n* = 27)	*P* value
LV function and strains				
LVEDV index (ml/m^2^)	58.1 ± 15.4	62.6 ± 11.5	61.1 ± 13.6	0.425
LVESV index (ml/m^2^)	25.8 ± 6.0	26.3 ± 6.5	25.6 ± 6.7	0.832
LVEF (%)	55.3 ± 4.5	56.5 ± 4.1	54.8 ± 5.1	0.527
LVM index (g/m^2^)	54.3 ± 4.2	55.3 ± 5.6	53.7 ± 5.2	0.659
LVGRS (%)	71.0 (61.8, 86.0)	76.9 (60.7, 94.8)	73.2 (62.1, 84.8)	0.871
LVGCS (%)	−20.2 ± 3.7	−20.9 ± 4.6	−22.0 ± 4.2	0.388
LVGLS (%)	−22.7 ± 3.7*	−21.2 ± 2.5	−19.6 ± 3.5	0.015
HDFStrength(RMS)-entire heartbeat				
HDF Strength(RMS)A-B(%)	19.0 (16.7, 22.6)	18.5 (15.7, 22.7)	18.7 (15.5, 23.7)	0.935
HDFStrength(RMS) L-S (%)	2.5 ± 0.7*	2.6 (2.1,3.8)	3.5 ± 1.2	0.036
LV- myo-LGE(%)	0 (0,8.6)	4.4 (0,7.8)	2.4 ± 0.7	0.102

* *P* value < 0.05 for vs. time ≥ 10y.

All abbreviations as in Table 2.

### Factors associated with HDF index in T2dm patients

Table 4 summarizes the univariate correlation coefficients of HDF Strength (RMS)A-B(%) and HDF Strength(RMS) L-S(%) with baseline clinical characteristics, extent of LGE, and myocardial strain in patients with T2DM.

**Table 4:** Univariable and multivariable linear regression analysis of all patients.

Variable	HDFStrength(RMS) A-B(%)				HDFStrength(RMS) L-S(%)			
	Univariable Multivariable				Univariable		Multivariable	
	*R* value	*P* value	*β* value	*P* value	*R* value	*P* value	*β* value	*P* value
Age (years)	0.066	0.608			0.145	0.258		
sex	0.018	0.886			−0.017	0.897		
bmi (kg/m^2^)	−0.021	0.870			0.225	0.076	0.140	0.220
SBP (mmHg)	0.059	0.696			0.084	0.515		
DBP (mmHg)	−0.181	0.156			0.222	0.865		
Diabetes duration (y)	−0.040	0.759			0.355	0.004	0.177	0.177
BUN (mmol/L)	0.115	0.368			−0.071	0.581		
Creatinine (μmol/L)	0.166	0.193			−0.016	0.903		
Total cholesterol (mmol/L)	−0.151	0.236			0.091	0.477		
Triglycerides (mmol/L)	0.063	0.625			0.178	0.163		
HDL-C (mmol/L)	−0.088	0.494			−0.018	0.887		
LDL-C (mmol/L)	−0.165	0.195			−0.054	0.672		
Hemoglobin A1C (%)	0.159	0.214			0.116	0.367		
Insulin	0.089	0.487			0.305	0.015	0.291	0.013
Metformin	0.025	0.843			0.087	0.498		
Sulphonylurea	−0.210	0.098	−0.214	0.076	−0.062	0.631		
Statin	−0.145	0.258			0.020	0.878		
Aspirin	−0.152	0.233			−0.003	0.982		
ACEI	0.235	0.064	0.006	0.964	0.084	0.511		
Diuretics	0.242	0.056	0.184	0.151	0.073	0.571		
Calciumchannelblockers	0.300	0.017	0.171	0.187	0.005	0.970		
β-blockers	−0.097	0.451			0.180	0.159		
LGE	−0.406	0.001	−0.329	0.006	0.531	<0.001	0.435	0.001
LVGLS	−0.019	0.884			−0.258	0.041	−0.210	0.057
LVGCS	−0.064	0.616			−0.059	0.644		
LVGRS	0.058	0.654			0.008	0.948		

Factors with *p* < 0.1 in the univariable analysis were included in the multivariable analysis.

All abbreviations as in Table 1 and Table 2.

In the T2DM patients, the HDF Strength (RMS)A-B(%) values were significantly associated with the LV- myo-LGE(%) (*r* = −0.406, *p* = 0.001). However, no significant associations were observed between the HDFStrength (RMS) A-B(%) values and the age, sex, BMI, blood pressure, DM duration, other biochemical indices, medications and myocardial strain. In the multivariable stepwise analysis, the independent determinant of the HD Strength (RMS) A-B(%) was the LV- myo-LGE(%) (*β* = −0.329, *p* = 0.006).

The HDF Strength(RMS)L-S(%) values were significantly associated with Diabetes duration, Insulin, the LV- myo-LGE(%) and myocardial strain (*r* = 0.355, *p* = 0.004; *r* = 0.305, *p* = 0.015; *r* = 0.531, *p* = 0.006; *r* = −0.258, *p* = 0.041). However, no significant associations were observed between the HDF Strength (RMS) L-S(%) values and the age, sex, BMI, blood pressure, DM duration, other biochemical indices and medications. In the multivariable stepwise analysis, the independent determinant of the HDF Strength (RMS) L-S(%) was the LV- myo-LGE(%) and insulin treatment (*β* = 0.435, *p* = 0.001; *β* = 0.291, *p* = 0.013) (Figure 3D).

### Intra-observer and inter-observer reproducibility

The intraclass correlation coefficient (ICC) values in the intraobserver analysis were 0.993, 0.943, 0.905, 0.957, 0.951 and 0.998 for LVGRS, LVGCS, LVGLS, HDF Strength(RMS)A-B(%), HDF Strength(RMS)L-S(%) and the extent of LGE, respectively. The ICC values in the interobserver analysis were 0.992, 0.928, 0.892, 0.948, 0.951 and 0.995 for LVGRS, LVGCS, LVGLS, HDF Strength(RMS)A-B(%), HDF Strength(RMS)L-S(%) and the extent of LGE, respectively.

## Discussion

Our present study demonstrated that (1) compared to the control group, LV function and myocardial strains were not significantly changed, but HDF Strength(RMS) L-S value was significantly higher in T2DM patients; (2) the longer the duration of diabetes, the more severe the damage of LVGLS, and the more obvious the damage of myocardial coordination in transversal directions HDF; (3) both longitudinal and main transversal directions HDF were significantly related to the extent of LGE.

Cardiovascular disease is the most important cause of mortality in diabetics and adults with diabetes have age-specific mortality rates that are four-fold greater than the general population (14). Therefore, it is necessary to detect diabetic cardiac physiology dysfunction as early as possible and to intervene clinically to improve the prognosis of patients. Nowadays, as cardiovascular imaging has made significant progress in quantitative assessment of cardiac function, HDF, as a relatively new imaging marker of LV function, is entering people's field of vision with great potential for clinical application.

Under normal conditions, the main HDFs are predominantly oriented along the basal-apical direction. Such an orientation optimizes the energy expenditure required to produce the stroke volume. Although the presence of a small transversal component is unavoidable due to the 3D anatomy, the appearance of relevant transversal components of HDF is always due to the breakdown of the delicate synergy/synchrony of the segmental myocardial deformation, which gives rise to abnormally oriented, transient pressure gradients. Thus, HDF Strength(RMS) L-S was significantly increased in T2DM patients in this study, indicating the coordination of myocardial diastolic and systolic motion were impaired. In addition, in this study, the synergy/synchrony transversal segmental myocardial motion is further impaired with the prolongation of diabetes duration. One possible reason is that long-term chronic hyperglycemia continuously causes myocardial cell damage and induces cell apoptosis by activating reactive oxygen species (ROS) production. The generation of ROS induces the formation of advanced glycation end products, eventually leading to myocardial fibrosis and remodeling, which produces a abnormally oriented, transient pressure gradients.

In this study, we found that patients with later stage DM had significantly impaired longitudinal strain compared with patients with early stage DM. However, Liu et al. announced that the difference in the longitudinal myocardial strain between newly diagnosed DM patients and longer-term DM patients was not significant (6). There are several common potential reasons for this difference, including patient demographics, and imaging techniques. Specifically, the number of patients in newly diagnosed DM group and longer-term DM group in the liu's study was roughly equivalent, but in our study, the number of patients with later stage diabetes was twice that of patients with early stage diabetes. Furthermore, the duration of diabetes was different between our study and liu's study. Although both his study and mine defined early diabetes as less than 5 years, our study defined the duration of later stage diabetes as more than 10 years, and Liu's was only more than 5 years. It is also possible that the imaging equipment is different, although the equipment is Siemens, but the imaging field strength is different. In view of the above reasons, there may be some differences between our study and liu's study. In our study, LV myocardial longitudinal strain was impaired in the later stage DM patients, which may be due to hemodynamic abnormalities. In short, the perturbation of IVPG leads to flow diversion, abnormal vorticity patterns, and ultimately impaired endocardial shear force (5). As we know, the myocardium consists of 3 layers (12, 15), and the orientation of LV subendocardial muscle fibers is longitudinal, therefore, GLS is initially abnormal when LV subendocardial shear stress is impaired. Studies have reported that there is an important relationship between HDF and segmental wall mechanics under physiological and pathological conditions (9, 16). In our study, HDF was correlated with LVGLS in the univariate correlation analysis, but there was no significant statistical significance in the multivariate correlation analysis, but there was a trend of correlation. If the sample size is increased, it may be meaningful. In the future study, we will continue to recruit patients with T2DM to further clarify the relationship between HDF and LVGLS.

LGE imaging on CMR is currently recognized as the gold standard for the identification of LV focal replacement fibrosis (17), and the extent of LGE can be quantified by professional post-processing software (18). In our current study, some T2DM patients have uneven patchy mild LGE in the middle of the myocardium, suggesting myocardial fibrosis and myocardial remodeling, and the extent of LGE were associated with longitudinal and transversal HDF values. The HDF represents the force exchange between ventricular blood and surrounding myocardium and is a global measure of the IVPG integrated over the LV volume (19). Alterations in HDF over the cardiac cycle indicate an alteration in blood-tissue interaction, possibly both a cause and consequence of the progression of structural remodeling. Multiple factors of myocardial remodeling have been reported in previous studies in DCM (20–22). Combining the LGE and HDF results of this study, HDF changes may also play an important role in triggering myocardial remodeling in DCM. Of course, further studies are needed to determine the sequence of myocardial remodeling and hemodynamic changes, and this is a rather promising study.

The recommended treatment for diabetes is usually a combination of drugs (12), which may include insulin. This study found a mild positive correlation between HDFStrength (RMS) L-S (%) and insulin. Several reasons may have contributed to this result. First, long-term chronic hyperglycemia directly causes myocardial cell damage and induces cell apoptosis, eventually leading to myocardial fibrosis and remodeling (23, 24). In this study, 33 patients (52.4%, a relatively high percentage) did not achieve ideal blood glucose levels despite long-term lifestyle changes and the use of antihyperglycemic agents. In this context, insulin was injected under the guidance of doctors according to individual differences until the blood glucose level was controlled within the normal range. During this process, long-term chronic hyperglycemia may lead to more serious myocardial damage (myocardial fibrosis/remodeling) than other T2DM patients. Second, insulin therapy may indirectly aggravate insulin resistance through a variety of factors, and further aggravate myocardial injury. These may lead to more destruction of synergy/synchrony transversal segmental myocardial motion. Of course, there was also a selection bias in this study. Whether insulin can promote transversal HDF damage still requires further study, and additional information is needed to determine whether there are correlations among the duration of insulin treatment, the order of insulin treatment and hemodynamics.

The assessment of LV function and the detection of mechanical abnormalities have undergone tremendous development from LVEF to deformation imaging. HDF is a novel imaging measure that provides the earliest signs of sub-clinical myocardial dysfunction even before strain and EF, which could complement existing diagnostic algorithms for early diabetic cardiac dysfunction. However, there are still potential barriers to implementing HDF analysis in routine clinical practice, such as the need for specialized software or expertise, but once implemented in routine clinical care, it can change the existing paradigm of cardiac function analysis. Therefore, it is necessary to increase relevant investment to achieve this goal.

## Limitation

In this study, several limitations should be considered. First, our sample size was relatively small (particularly among patients with diabetes duration of less than 5 years), which leads to potential bias.The limited number of participants reduce the generalizability of findings or increase the risk of Type II errors. In future studies, we will conduct multicenter or longitudinal cohort studies to overcome this limitation. Second, in this study, we recruited patients with type 2 diabetes who did not have hypertension, which limits the applicability of findings to typical clinical populations with T2DM, where hypertension is prevalent. In the future, we will conduct studies including hypertensive patients to validate and extend the findings. Third, there are some imaging techniques related to any potential limitations, such as the dependency on cine-CMR quality or variability in post-processing software. Fourth, exclusion of other coexisting conditions (obesity, dyslipidemia) would have narrowed the scope of the study further. Fifth, reliance on medical history and examination reports from six months prior may overlook interim health changes, potentially misclassifying controls. We will continue to recruit volunteers with complete real-time biochemical measurements for the next phase of our study. Sixth, insulin treatment is associated with HDF Strength(RMS) L-S, the study does not explore potential confounders, such as differences in diabetes severity or adherence to treatment regimens, that might influence this finding.

## Conclusions

HDF analysis can detect the delicate synergy/synchrony destruction of segmental myocardial motion before the abnormality of LV ejection fraction and GLS in T2DM patients. HDFs parameters are associated with the extent of LGE, and the synergy/synchrony transversal segmental myocardial motion is further impaired with the prolongation of diabetes duration. HDF analysis has the potential to add incremental clinical value, allowing earlier detection of pathology or immediate evaluation of response to treatment.

## Data Availability

The original contributions presented in the study are included in the article/Supplementary Material, further inquiries can be directed to the corresponding authors.
